# Clinical Impact of NOTCH3 Variant Location After First Stroke in CADASIL

**DOI:** 10.1002/acn3.70424

**Published:** 2026-05-06

**Authors:** Léa Aguilhon, Hugues Chabriat, Dominique Hervé, Stéphanie Guey, Sophie Tezenas Du Montcel, Juliette Ortholand

**Affiliations:** ^1^ Sorbonne Université, Institut du Cerveau—Paris Brain Institute—ICM, CNRS, Inria, Inserm, AP‐HP, Hôpital de la Pitié Salpêtrière Paris France; ^2^ GENOVASC, INSERM U1127 Paris Brain Institute (Institut du Cerveau—ICM) Paris France; ^3^ Centre de Référence National des Maladies Rares des Vaisseaux du Cerveau et de L'œil (CERVCO) and Centre Neurovascular Translationnel (CNVT), FHU‐NeuroVasc‐2030 Hopital Lariboisiére APHP Paris France; ^4^ Université Paris‐Cité Paris France

**Keywords:** CADASIL, NOTCH3, principal stratification, stroke

## Abstract

**Objective:**

Despite its monogenic origin, Cerebral Autosomal Dominant Arteriopathy with Subcortical Infarcts and Leukoencephalopathy exhibits marked variability in clinical expression and severity. Variants in the NOTCH3 gene, within epidermal growth factor‐like repeat domains 1–6 or 7–34, are known to influence disease onset, but their impact on long‐term progression remains unclear. This study assesses mutation location effects on post‐first stroke clinical trajectories.

**Methods:**

Clinical data from a large cohort were analyzed (Patients EGFR 1–6 mutation group *n* = 210 and 7–34 mutation group *n* = 116) with target emulated trial framework. To study the impact of mutation location on stroke recurrence, disability (modified Rankin score ≥ 3) and mortality, following a first stroke event. Propensity score matching was used to balance covariates between mutation location groups and principal stratification to consider truncation by death. Events occurrence differences were compared using Restricted Mean Survival Time at 2, 5, 10 and 15 years.

**Results:**

At first stroke, patients with mutation in domains 1–6 were younger than those in the 7–34 mutation group (49.51 ± 7.4 vs. 55.00 ± 7.4 years). Ten years after first stroke event, mortality occurred slightly later in the 7–34 group (9.63 [9.33–9.92] vs. 9.11 [8.71–9.52] years, *p* = 0.04), also at 15 years (14.0 [13.42–14.63] vs. 12.4 [11.62–13.24] years; *p* = 0.002). Second stroke occurrence did not differ between groups. Time beyond modified Rankin of 3 slightly differed between groups at 5 and 10 years, with a difference of 0.22 [0.01–0.044] and 0.72 [0.14–1.30] year respectively (*p* = 0.044 and 0.017).

**Interpretation:**

Although NOTCH3 variants location influences the delay to the first stroke, it has no or little impact on the recurrence of stroke, risk of disability and death after the first stroke manifestation.

## 1 Introduction

Cerebral Autosomal Dominant Arteriopathy with Subcortical Infarcts and Leukoencephalopathy (CADASIL) is the most frequent hereditary small vessel disease (cSVD) leading to stroke and dementia [1, 2]. The disease is caused by cysteine pathogenic variants of the NOTCH3 gene [3, 4] and is easily diagnosed worldwide by genetic testing. CADASIL leads to a large spectrum of clinical manifestations [2]. Strokes resulting from small, deep ischaemic lesions whose accumulation progressively drives motor and cognitive decline represent a key manifestation of the disease [5, 6]. However, despite its monogenic origin, this genetic condition displays marked interindividual heterogeneity [7, 8]. Age at onset, symptom severity, clinical manifestations, and disease course vary substantially among patients [7, 9, 10].

In recent years, the location of cysteine mutations within the epidermal growth factor‐like repeat (EGFr) domains of the NOTCH3 gene has been shown to play a crucial role in determining disease severity. Population‐based studies have revealed an unexpectedly high frequency of completely asymptomatic or only mildly symptomatic individuals carrying mutations in EGFr domains 7–34 [11, 12, 13]. In hospital cohorts, individuals with mutations in EGFr domains 1–6 typically present with a more severe phenotype and represent the subgroup most frequently diagnosed in clinical settings [7, 14]. Finally, recent data indicate that the accumulation of NOTCH3 protein may vary according to the mutation site, with higher levels observed in patients harboring cysteine mutations in EGFr domains 1–6 than in those carrying mutations in other EGFr domains [13].

However, although the influence of mutation location is well established for age at first stroke and for cross‐sectional comparisons of disease severity within patient cohorts, presumably reflecting differential accumulation of mutant NOTCH3 protein in the vascular wall [15], whether disease progression after stroke onset also differs according to the mutation site remains uncertain. We aim to address this question, which is crucial in preparing for future clinical trials.

However, this entails two principal challenges. The first is the identification of a CADASIL cohort with a detailed mutation classification and a sufficiently long prospective follow‐up to capture clinically meaningful outcomes. Second, an appropriate analytical framework is needed, as conventional observational approaches are limited in their ability to quantify variant‐specific effects owing to potential residual confounding and selection biases. Analyses of time‐to‐event outcomes, such as recurrent stroke, are further complicated because of death, as a competing risk. As once patients die, they can no longer experience subsequent events, which distorts estimates and obscures causal pathways.

To address these challenges, (1) we analyzed disease trajectories in a large cohort of patients with NOTCH3 mutations in EGFr domains 1–6 or 7–34 who were followed prospectively over an extended period; (2) focused on post‐first‐stroke progression and estimated the risks of recurrent stroke, developing moderate‐to‐severe disability, and death; (3) we balanced the two groups using propensity score matching to limit confounding bias, and (4) we estimated these risks within a causal inference framework called Principal Stratification, which can model potential survival outcomes under alternative scenarios, thereby accounting for truncation by death and reducing bias related to survival time.

## 2 Methods

### 2.1 Study Population

Observational data were collected from patients enrolled at the French National Referral Center for rare cerebrovascular diseases (CERVCO, www.cervco.fr). They were analyzed after obtaining written consent from all participants and ethical approval. The study was approved by an independent ethics committee from INSERM (CEEI‐IRB‐17/388). All participants had a confirmed diagnosis of CADASIL through genetic testing, which showed an archetypal cysteine variant in the NOTCH3 gene. Patients were consecutively recruited between 2003 and 2024 and followed up regularly when they agreed to return to the center or were contacted by telephone. Data were recorded in a REDCap database. From the overall cohort, we selected patients who had at least one ischemic or hemorrhagic stroke before or after inclusion in the study. Visits are planned regularly every 2 years and are not dependent on routine follow‐up.

### 2.2 Outcomes and Covariates

The analysis was structured around a comparison between patients harboring NOTCH3 mutations in EGFr domains 1–6 and those with mutations in domains 7–34 [16, 17, 18]. The events considered for group comparisons included the occurrence of a second stroke, reaching a modified Rankin score (mRS) ≥ 3, and death, with time‐to‐event measured in years from the first stroke.

In the analysis, five patients who died from their second stroke were classified as having a stroke before their death. Therefore, they were first considered to belong to the stroke group. Subsequently, a sensitivity analysis was performed by reclassifying these cases into the mortality group to evaluate the impact of such categorization.

Other covariates included age at first stroke, sex (male/female), education level (years in school ranging from 0 to 22), and cardiovascular risk factors, including hypertension (present/absent), hypercholesterolemia (present/absent), alcohol consumption (yes/no), and smoking status (current or former/never). These different variables were considered based on their established or potential association with accelerated disease progression, in addition to the mutation location site [10, 19]. They were recorded as closely as possible to the first stroke event and allowed deriving a propensity score used to pseudo‐randomize patients. The age at the time of the first stroke was also compared between the groups.

### 2.3 Statistical Analysis

We use the target trial emulation framework to conduct that study following the TARGET guideline [20]. The related materials can be found in appendix (Table 1; Table S1).

**TABLE 1:** Target trial protocol and observational emulation: Specification of the protocol of the target trial and its observational emulation.

	Target trial	Observational emulation
Eligibility criteria	CADASIL disease First stroke	CADASIL disease First stroke Enter cohort between 2003 and 2024
Treatment strategies	Change the NOTCH3 mutation	Change the NOTCH3 mutation
Assignment procedures	Randomized	Randomized
Follow‐up	2, 5, 10, 15 years	2, 5, 10, 15 years
Outcome(s)	Rankin, second stroke, survival	Rankin, second stroke, survival
Causal contrast(s)	RMST, with principal stratification for rankin and second stroke as they can be censored by death	ATT with RMST, with principal stratification for rankin and second stroke as they can be censored by death
Identifying assumptions		**SUTVA (Stable Unit Treatment Value Assumption):** we assume that selecting patient from first stroke adjust for disease severity, and we adjust for additional covariates: age at first stroke, sex, education level, and cardiovascular risk factors, including hypertension, hypercholesterolemia, alcohol consumption, and smoking status. **Positivity:** We use propensity score matching with for treatment group [Egfr 1–6]. **NUC (No Unmeasured Confounder):** The disease is genetic and we have tried to correct for both disease severity and main habits that influence the disease progression.
Data analysis plan	Prediction of always survivors RMST, with principal stratification for mRS and second stroke	Propensity score matching Prediction of always survivors RMST, with principal stratification for mRS and second stroke

#### 2.3.1 Cohort Description and Adjustment for Potential Confounding Effects at Time of the First Stroke Event Before Group Comparisons

Baseline characteristics and age at first stroke were compared between groups using non‐parametric Wilcoxon tests for continuous variables and Fisher tests for categorical variable measures, with a significance threshold set at α = 0.05.

In the present study, the age at the time of the first stroke was used as the time reference for each patient. All selected covariates were included in the matching procedure to generate a balanced subpopulation for subsequent analyses. This analytical framework, originally developed for therapeutic evaluation in clinical research (MatchIt R package [21] and Cobalt R package [22]), was adapted here for observational data to estimate the average effect among individuals in the 1–6 mutation group, considered as the reference population. Following matching, we assumed that individual outcomes were independent (no interference between subjects), that all groups had comparable probability of assignment (positivity), and that no key confounders were omitted, as required under target trial emulation principles [20].

#### 2.3.2 Group Comparison of Survival Curves Death Since the First Stroke Event

Mortality analyses were first performed using the Kaplan–Meier estimation. The log‐rank test (R package nph [23]) was used to estimate the differences in the overall survival curves between the two groups of patients.

Mortality was also analyzed by computing the Restricted Mean Survival Time (RMST) [24] at 5, 10, and 15 years after the first stroke event. This non‐parametric approach was used to quantify the area under the survival curve up to these different specified time horizons and to provide estimates of survival time differentials while avoiding proportional hazard assumptions.

#### 2.3.3 Group Comparison of Event‐Free Probability Curves for the Occurrence of a Second Stroke or mRS ≥ 3 After the First Stroke Event

Principal stratification [25] was employed to account for death as a competing event of the second stroke. It allows a causal inference analysis of outcomes (second stroke and mRS ≥ 3), only in an always survivor group. To do so, patients were classified into four latent groups based on: (1) their real survival status (observed outcome), and (2) their counterfactual survival status (predicted outcome if they had the opposite mutation of what they actually have). The Always Survivors subgroup (survival under both mutation types) was selected and analyzed to isolate mutation effects while addressing mortality bias [25] for each time point.

Counterfactual event‐free probability was predicted using a proportional hazards model (Cox model) (Survival R package [26, 27]) with a 0.5 probability threshold to define the event‐free status. The accuracy of probabilistic predictions was tested using two metrics: the cumulative Area Under the Curve (AUC) to measure the quality of ranking (order) in predictions [28] (worst 0.5—best 1); and the Integrative Brier Score to quantify the accuracy through an approximate distance between the predicted and actual outcome [29] (worst: 1; best: 0).

Differences between the two mutation location groups in the occurrence of a second stroke and mRS ≥ 3 were assessed using RMST analysis at 2‐, 5‐, 10‐, and 15‐year intervals after the first stroke event. The survRM2 R package [30] was used for this analysis (estimates and confidence intervals were computed). These different time points were selected to capture the various durations of disease courses from the first stroke event.

In addition to right censoring, mRS data were left and interval censored. Even though the visit scheme was fixed and should not depend on patient health, we assessed whether the two censoring mechanisms depend on the baseline covariates. A sensitivity analysis was also performed by computing RMST, assessed at the beginning, the midpoint, and the end of each interval, to evaluate the robustness of the findings. We also performed a sensitivity analysis using the same statistical framework, for comparing High (EGFr 1–6, 8, 11 and 26) and Mid‐Risk (EGFr 9, 10, 12–15, 17, 25, 27 and 32) EGFr domain, based on EGFr risk stratification proposed by Hack et al. [17]. The analysis of the Low‐group was not possible due to limited statistical power (small sample size and number of events).

## 3 Results

### 3.1 Study Population

Data were collected from a total of 536 patients; we then selected 326 patients in this cohort population who already had stroke manifestations (Table 2; Table S2). Individuals with a mutation located in EGFr domains 1–6 (later called group 1–6) were significantly younger at their first stroke (median 49.5 (IQR: 42.1–56.9)) than those with a mutation located in EGFr domains 7–34 (later called group 7–34) (median 55.0 (47.7–62.4), *p* value < 0.001). They also had less frequent hypertension and hypercholesterolemia than patients with a mutation in the 7–34 EGFr domains of the NOTCH3 gene (Table 2).

**TABLE 2:** Comparison of 1–6 and 7–34 EGFr mutations in the initial stroke population.

Variables	1–6 (*n* = 210)	7–34 (*n* = 116)	*p* value
Male gender (*n* (%))	122 (58%)	55 (47%)	0.081
**Age at first stroke (Median (Q1‐Q3))**	**49.5 (42.1–56.9)**	**55.0 (47.7–62.4)**	**< 0.001**
Nb years of education (Median (Q1–Q3))	11.0 (9.0–14.0)	10.0 (9.0–14.0)	0.935
**Hypertension (*n* (%))**	**47 (22%)**	**53 (46%)**	**< 0.001**
**Hypercholesterolemia (*n* (%))**	**86 (41%)**	**63 (54%)**	**0.027**
Smoking (*n* (%))	110 (52%)	(46%)	0.298
Alcohol (*n* (%))	117 (56%)	65 (56%)	> 0.999

*Note:* Bold values indicate statistically significant results (*p* ≤ 0.05).

### 3.2 Adjustment Before Comparison Between Group 1–6 and Group 7–34

**FIGURE 1:**
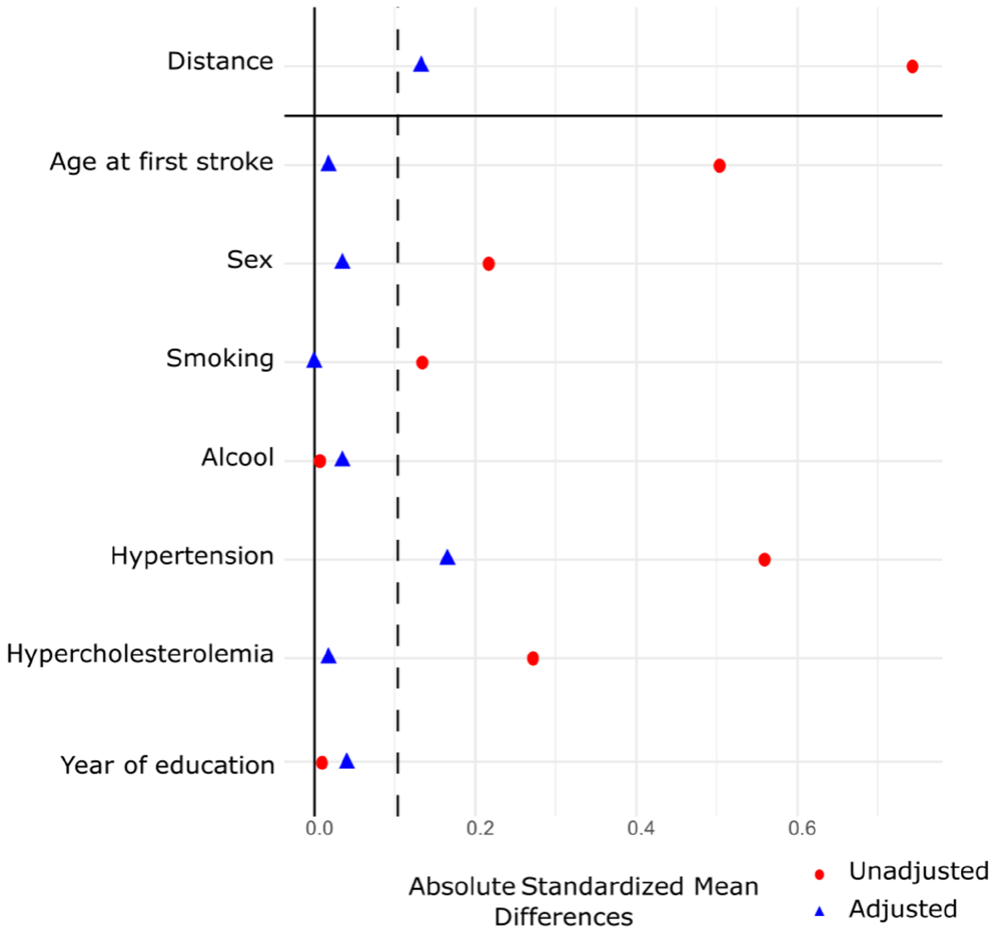
Balance assessment between mutation location in EGFR domains (1–6 vs. 7–34) of potential confounding factors before and after adjustment using propensity score matching method.

The adjustment performed using propensity score methods to minimize any potential confounding effect was effective. Five variables were initially unbalanced between the two groups (sex, smoking status, hypertension, hypercholesterolemia, and age at first stroke) (Figure 1) with age at first stroke and hypercholesterolemia exhibiting the largest disparity (Absolute Standard Mean Difference = −0.51 and −0.56). After the balancing, only hypertension remained slightly unbalanced between the two groups, but no statistical difference was detected between the two pseudo‐randomized groups. Finally, the procedure allowed matching 116 individuals from the 1–6 group to 116 individuals from the 7–34 group. This selection of patients was then used for the following analysis and the subselection of “always survivors” groups.

### 3.3 Comparison of Probability Survival of the Death Between Group 1–6 and Group 7–34

**FIGURE 2:**
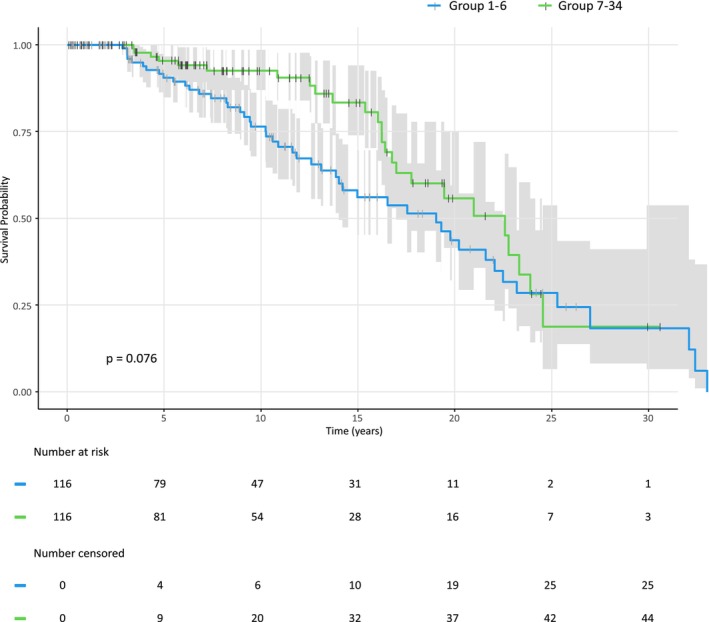
Probability analysis (Kaplan–Meier) of survival by mutation location in EGFR domains (1–6 vs. 7–34).

The comparison of survival curves between the two groups was performed in the pseudo‐randomized subpopulation (232 patients). On the full range of follow‐up, no statistically significant difference was detected (median = 19.03 [14.01–22.47] years and 22.58 [16.97–NA]) for the 1–6 group and 7–34 group, respectively (Log‐rank test, *p* value = 0.08) (Figure 2).

When considering specific time‐points, analysis of the restricted mean survival time (RMST) did not show any significant difference between the two groups at 5 years (*p* value = 0.1) but revealed a lower survival in the 1–6 group at 10 years (RMST of 9.11 years [8.71–9.51] vs. 9.63 years [9.33–9.92] (*p* value = 0.040)) and at 15 years of follow‐up (RMST of 12.4 years [11.62–13.24] vs. 14.0 years [13.42–14.63] (*p* value = 0.002)) (Table 3).

**TABLE 3:** RMST comparison of death between EGFR mutation (1–6 vs. 7–34) at 5, 10, and 15 years post‐first stroke; and of second stroke and mRS > 3, between EGFR mutation (1–6 vs. 7–34) at 2, 5, 10, and 15 years post‐first stroke in distinct “always‐survivor” subgroups (i.e., patients surviving at least to each timepoint).

Years to predict	Nb (always survivors)	RMST 1–6 mutation	RMST 7–34 mutation	Difference	*p* value
Death					
5 years	—	4.88 [4.79–4.97]	4.95 [4.90–5.00]	−0.08	0.143
**10 years**	—	**9.11 [8.71–9.52]**	**9.63 [9.33–9.92]**	**−0.52**	**0.040**
**15 years**	—	**12.43 [11.62–13.24]**	**14.03 [13.42–14.63]**	**−1.60**	**0.002**
Second stroke					
2 years	232	1.75 [1.66–1.85]	1.73 [1.62–1.85]	0.02	0.795
5 years	216	3.96 [3.62–4.29]	3.90 [3.56–4.24]	0.06	0.814
10 years	195	6.67 [5.83–7.53]	6.55 [5.76–7.34]	0.13	0.830
15 years	154	8.85 [7.31–10.39]	8.74 [7.39–10.08]	0.11	0.916
Rankin > 3					
2 years	232	1.93 [1.87–1.99]	1.99 [1.96–2.00]	0.06	0.094
**5 years**	**216**	**4.72 [4.52–4.92]**	**4.94 [4.86–5.03]**	**0.22**	**0.044**
**10 years**	**195**	**9.03 [8.51–9.55]**	**9.75 [9.48–10.01]**	**0.72**	**0.017**
15 years	154	13.32 [12.34–14.30]	14.02 [13.35–14.68]	0.69	0.252

*Note:* Bold values indicate statistically significant results (*p* ≤ 0.05).

### 3.4 Comparison of Probability Survival of a Second Stroke Between Group 1–6 and Group 7–34

**FIGURE 3:**
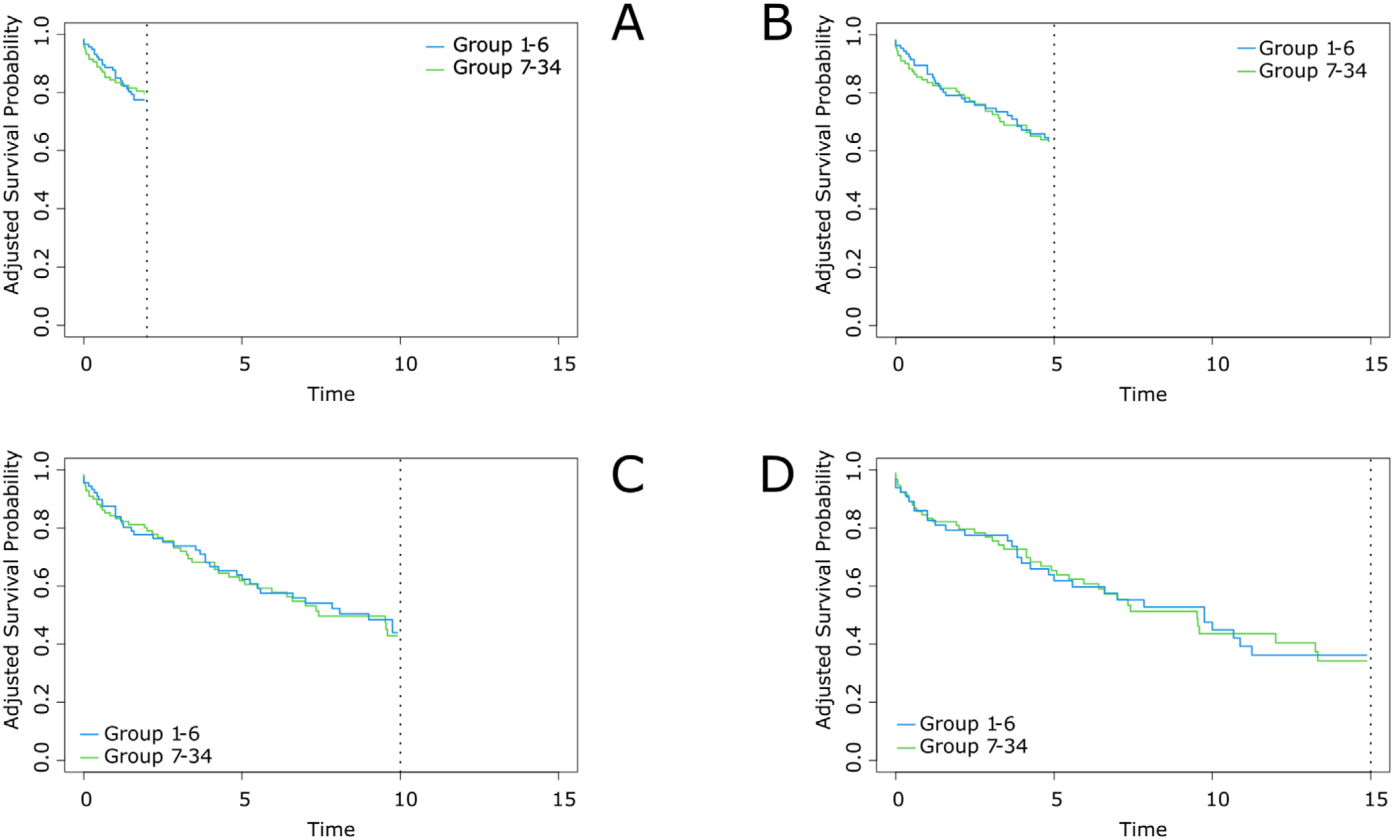
Probability analysis (Kaplan–Meier after adjustment) of survival without a second stroke with time elapsed since 2 (A), 5 (B), 10 (C), and 15 (D) years after the first stroke event, in distinct “always‐survivor” subgroups (i.e., patients surviving at least to each timepoint), stratified by EGFR mutation domain (1–6 vs. 7–34).

The prediction cox model of death used to estimate the subpopulation of “always survivors” (event‐free population) (Supplementary Tables S3–S6 and Figures S1–S4) after their first stroke showed good performances with a cumulative AUC and Integrative Brier Score of 0.84 and 0.32, respectively. Then, in this subpopulation, the Kaplan–Meier curves were found to be superimposed for the four follow‐up durations (*p* values always > 0.05) (Figure 3), with very small and non‐significant differences in RMST between the two groups (0.038, 0.099, 0.228, and 0.116 at respectively, 2, 5, 10, and 15 years of follow‐up after the first stroke event) (Table 3). In the sensitivity analysis, patients who died from stroke (*n* = 5), initially classified as “stroke events” in the primary analysis, were reclassified as “deaths”. This modification did not alter the results, confirming the robustness of our findings (Figure S5).

### 3.5 Comparison of Probability Survival of a mRS ≥ 3 Between Group 1–6 and Group 7–34

For the analysis of disability, as the data are interval‐censored, we first evaluated potential bias relative to censoring. First, left censoring can be considered as non‐informative, because only a few patients are censored (around 10%), and baseline characteristics did not differ between censored and non‐censored patients (Table S7). We also analyzed the distribution of censoring interval durations (i.e., time between visits) across covariates, which did not reveal a significate group difference (Figure S6). Finally, given that patients had a visit every 2 years, interval censoring can be considered as not informative and is relatively balanced across patients.

**FIGURE 4:**
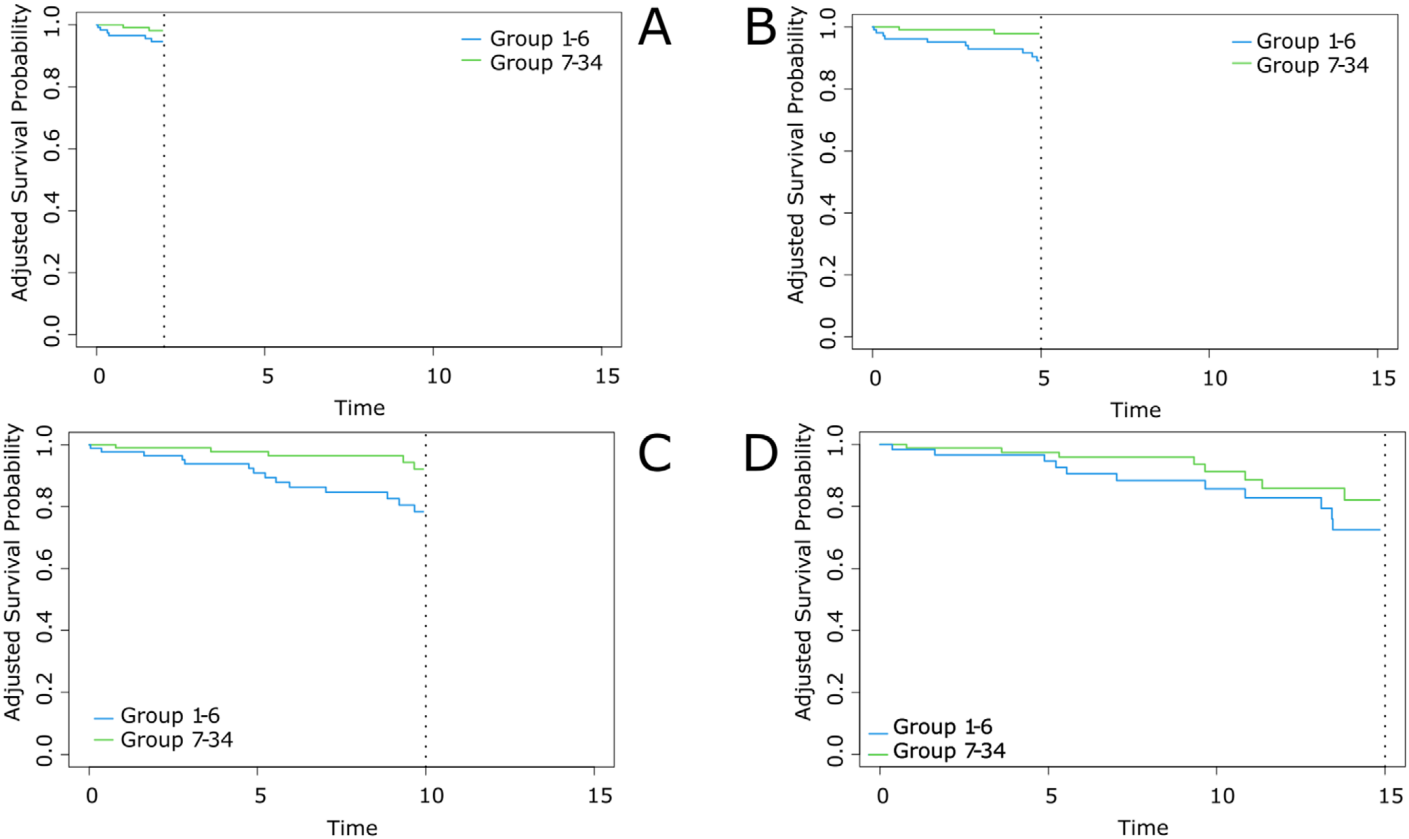
Probability analysis (Kaplan–Meier after adjustment) of survival without reaching mRS ≥ 3 with time elapsed since 2 (A), 5 (B), 10 (C), and 15 (D) years after the first stroke event, in distinct “always‐survivor” subgroups (i.e., patients surviving at least to each timepoint), stratified by EGFR mutation domain (1–6 vs. 7–34).

The RMST up to the observation of mRS ≥ 3 with repeated clinical evaluation (Table 3, Figure 4) was slightly smaller in group 1–6 than in the other group at 5 years (4.72 [4.52–4.92] years vs. 4.94 [4.86–5.03], *p* = 0.04) and 10‐year (9.03 [8.51–9.55] vs. 9.75 [9.48–10.01] years, *p* = 0.017). In contrast, no significant differences were detected at the 2‐year or 15‐year horizons. Regarding interval censoring, we performed the RMST analysis at the start, the middle, and the end of the interval, with similar results (Table S8).

### 3.6 Sensitivity Analysis for High and Mid‐Risk EGFr Domain Comparison

Analysis was therefore conducted comparing the High‐ and Mid‐risk EGFr groups (218 vs. 83 patients; 121 vs. 36 events). Analyses of death, recurrent stroke, and disability (mRS) showed slightly larger differences between these groups than those observed with the 1–6 versus 7–34 classification, without any meaningful change in statistical significance. The pattern remained heterogeneous over time, with no consistent trend across outcomes (Table S9), in line with the overall results.

## 4 Discussion

The time‐related risk of recurrent stroke after a first clinical event in CADASIL patients does not differ between individuals carrying mutations in EGFr domains 1–6 and those with cysteine mutations in other EGFr domains. This absence of difference persisted throughout follow‐up, up to 15 years after the first stroke. These results are in contrast with the age at first stroke that differed markedly between the two groups selected for analysis, with a gap of 5.5 years mean difference in line with previous results indicating that individuals with a cysteine mutation in EGFr 1–6 have earlier and more severe phenotypic manifestations of the disease after correction for age [7, 14, 16, 31]. Altogether, our findings suggest that while mutation location strongly influences the timing of the initial ischemic manifestation, it may have little impact on disease evolution thereafter. These negative findings, specifically focused on the disease course after the occurrence of stroke, do not necessarily contradict results evaluating progression in cohorts over a limited follow‐up and including both presymptomatic and symptomatic individuals or relied on multiple clinical and imaging endpoints [16], as the studies addressed different phases of the disease.

A possible explanation is that the first stroke occurs once a threshold of NOTCH3 protein accumulation in the vascular wall has been reached, since accumulating evidence supports that mutation location influences the level of extracellular NOTCH3 deposition, as demonstrated in vessels of patients with skin biopsies [15]. Conversely, beyond this stage, the course of the disease, and particularly the risk of further ischemic events, might be driven predominantly by other mechanisms. Experimental evidence indicates that loss of smooth muscle cells and progressive fibrosis develop after sustained NOTCH3 extracellular domain accumulation [32]. It is therefore plausible that these structural alterations or other factors play a greater role in advanced stages of the disease than the accumulation of the NOTCH3 protein itself [33, 34].

When examining the occurrence of moderate‐to‐severe disability after the first stroke in our cohort, we observed a slight difference between patients with mutations in EGFr domains 1–6 and those with mutations in domains 7–34. This difference was not statistically significant when follow‐up was restricted to 2 or 15 years, but it reached significance at 5‐ and 10‐year follow‐up. On average, the delay to disability differed by only a few months to less than 1 year, suggesting that moderate‐to‐severe disability may occur slightly earlier in the 1–6 group after the first stroke has occurred. This modest difference nevertheless still contrasts with the much larger gap in age at first stroke between the two groups. Unlike the stroke event itself, which is time‐limited and precisely dated, disability progression reflects however much more the cumulative burden of cerebral lesions that may not exclusively depend on the accumulation of focal ischemic events [6]. These findings should therefore be interpreted with caution, particularly since the Rankin score was assessed only every 2 years in our study, precluding determination of the exact date of disability progression. In addition, because we did not have information on the Rankin score immediately after the first stroke, we cannot exclude the possibility that patients with mutations in EGFr domains 1–6 were already slightly more disabled at stroke onset, which may have also contributed to the subsequent risk of disability worsening.

Our results also show that the risk of death after the first stroke does not differ by mutation location at 5 years of follow‐up. However, at 10 and 15 years, a slight difference emerges, corresponding to an average of 1–2 years. These findings are consistent with the 2‐ to 3‐year shift according to mutation location observed in the analysis of multiple disease course parameters within the overall cohort [10]. They further suggest that, beyond the occurrence of the first stroke, differences in risk and survival related to mutation location remain limited, and that once a certain threshold is reached, the disease tends to evolve in a broadly similar manner across individuals.

We believe the results of this study are robust for several reasons. The use of propensity score matching reduced bias from confounding covariates and approximated the conditions of a randomized controlled trial. In addition, the application of principal stratification allowed us to disentangle the effect of mutation location on recurrent stroke risk from its effect on mortality, thereby minimizing bias related to competing risks, a frequent challenge in cerebrovascular disease research. This analytical framework strengthens the reliability of our genetic effect estimates. Another important strength is the use of a large database, which provides clinically relevant longitudinal information on the disease course in a detailed‐characterized cohort.

Nevertheless, several limitations must be acknowledged. First, we focused exclusively on patients with clinically recognized stroke, which has excluded individuals who experienced atypical or silent ischaemic cerebral lesions. Second, we did not include longitudinal disability scores in the analyses because of insufficient data, although such measures might have provided further insight into disease progression across mutation groups. Third, inherent biases linked to the cohort itself and to recruitment strategies cannot be entirely excluded, as discussed earlier, such as interval censoring. Moreover, regarding the no unmeasured confounder assumption, age‐related brain factors such as the brain reserve at the time of the first stroke event could not be accounted for and may not have been fully mitigated by the statistical approaches we used.

Another limitation of this study is that we could not apply the total refined three‐tier NOTCH3 variant classification proposed by Hack et al. [17] to our data, as several variant categories were insufficiently represented aftermatching and event‐based restrictions, precluding adequately powered comparisons. However, a sensitivity analysis comparing high‐ versus moderate‐risk groups did not show any consistent and robust pattern of association over time, which did not challenge the overall findings of the main analysis. Larger cohorts will be required to determine whether any stratification could provide incremental prognostic value in CADASIL.

In summary, although cysteine NOTCH3 mutation location in EGFr domains has a major impact early in CADASIL by determining the age at first stroke, our findings indicate that disease progression after this event is broadly similar across mutation groups. This novel observation may have important implications for therapeutic evaluation and risk stratification.

## Author Contributions

Léa Aguilhon contributed to the study design, data analysis, statistical modeling, figure preparation, and drafting of the manuscript. Juliette Ortholand and Sophie Tezenas Du Montcel supervised the work, contributed to the study conceptualization and design, and participated in manuscript writing and revision. Hugues Chabriat contributed to manuscript writing and critical revision. Dominique Hervé and Stéphanie Guey were responsible for data acquisition. All authors reviewed and approved the final version of the manuscript.

## Funding

This study is supported by grants from the French Ministry of Health (Regional and National PHRC AOR 02‐001) and Research (Agence National de la Recherche, ANR, RHU TRT_cSVD), Association de Recherche en NEurologie VAsculaire, and from the European Joint Program for Rare Disease—2024 of Horizon H2020 (CADANHIS) of Agence Nationale de la Recherche as part of the program (reference ANE‐23‐RAR4‐0002). The research leading to these results has received funding from the French government under management of Agence Nationale de la Recherche as part of the “Investissements d'avenir” program (reference ANR‐19‐P3IA‐0001, PRAIRIE 3IA Institute).

## Conflicts of Interest

Hugues Chabriat has received research funding from ARNEVA (Association de Recherche en Neurologie Vasculaire) since the initial planning of the work. He also reports grants from the Agence Nationale de la Recherche (ANR) for the project ANR TR–cSVD and from the European Joint Programme on Rare Diseases (EJPRD) for the project CADANHIS. The remaining authors declare that they have no competing interests.

## Supporting information


**Figure S1:** Principal stratification classification at 2 years after first stroke.
**Figure S2:** Principal stratification classification at 5 years after first stroke.
**Figure S3:** Principal stratification classification at 10 years after the first stroke.
**Figure S4:** Principal stratification classification at 15 years after first stroke.
**Figure S5:** Sensitivity analysis: Adjusted Kaplan–Meier estimates of stroke‐free survival from 2 (A), 5 (B), 10 (C), and 15 (D) years after first stroke, in “always‐survivor” subgroups, classifying as deaths (death prioritized over stroke), stratified by EGFR mutation domain (1–6 vs. 7–34).
**Figure S6:** Length of the interval censoring for Rankin analysis, depending on baseline covariates.
**Table S1:** TARGET Guideline checklist.
**Table S2:** Patients number by EGFr domain before and after matching.
**Table S3:** Population description of the “always survivors” group at 2 years after first stroke.
**Table S4:** Population description of the “always survivors” group after 5 years after the first stroke.
**Table S5:** Population description of always survivors at 10 years after first stroke.
**Table S6:** Population description of always survivors at 15 years after first stroke.
**Table S7:** Comparison of baseline characteristics between patients left censored or not for mRS > 3.
**Table S8:** Sensitivity analysis for censoring by interval for mRS analysis: RMST analysis using the start and the middle of the interval as event date.
**Table S9:** RMST Comparison of Death Between EGFR Mutation (High‐Risk vs. Mid‐Risk) at 5, 10, and 15 Years Post‐First Stroke; and of Second Stroke and mRS > 3, between EGFR Mutation (High‐Risk vs. Mid‐Risk) at 2, 5, 10, and 15 Years Post‐First Stroke in Distinct “always‐survivor” Subgroups (i.e., patients surviving at least to each timepoint).

## Data Availability

The data that support the findings of this study are available on request from the corresponding author. The data are not publicly available due to privacy or ethical restrictions. Code in R for study design and analysis are available on request on GitHub repository: https://github.com/lea‐aguilhon/CADASIL_Principal_Stratification.
