# Supplementary material for: Clinical Impact of NOTCH3 Variant Location After First Stroke in CADASIL
Source: Ann Clin Transl Neurol. 2026 May 6:10.1002/acn3.70424. Online ahead of print. doi: 10.1002/acn3.70424 (PMC13394065; doi:10.1002/acn3.70424)

***SUPPLEMENTARY MATERIAL***

**Table S1: TARGET Guideline checklist**

| **Item no.** | | | | **Checklist item** | | | | | **Location reported** | |
| --- | --- | --- | --- | --- | --- | --- | --- | --- | --- | --- |
| **Abstract** | | | | | | | | | | |
| **1** | a | | | | Identify that the study attempts to emulate a target trial using observational data. State the study objectives and briefly summarize the specified target trial. | | | | §2 method section | |
|  | b | | | | Report the data sources used for emulation. | | | | §2 method section | |
|  | c | | | | Summarize key assumptions, statistical methods, findings and conclusions. | | | | §2 and §3 | |
| **Introduction** | | | | | | | | | | |
| **2** | Background | | | | Describe the scientific background of the study and the gap in knowledge. | | | | p.3 § 2 | |
| **3** | Causal question | | | | Summarize the causal question. | | | | p.3 §3 | |
| **4** | Rationale | | | | Describe the rationale for emulating a target trial with the available data. Cite randomized trials informing the design of the target trial if applicable. | | | | Non Applicable | |
| **Methods** | | | | | | | | | | |
| **5** | Data sources | | Cite the data sources contributing to the analyses and for each one describe the following: original purpose, type, the geographic locations, setting and time-period. If relevant, describe how the data were linked or pooled. | | | | | | p.6: Study population section | |
| **6** | **Target trial specification**  Specify the components of the target trial protocol that would answer the causal question. | | | | | **7** | **Target trial emulation**  Describe how the components of the target trial protocol were emulated with the observational data, including how all variables were measured or ascertained. | | **Location item 6 (specification) reported** | **Location item 7 (emulation) reported** |
|  | **Eligibility criteria** | | | | |  | **Eligibility criteria** | |  |  |
|  | a | Describe the eligibility criteria. | | | |  | a | Describe how the eligibility criteria were operationalized with the data. |  | p.5: Study population section |
|  | **Treatment strategies** | | | | |  | **Treatment strategies** | |  |  |
|  | b | Describe the treatment strategies that would be compared. | | | |  | b | Describe how the treatment strategies were operationalized with the data. |  | p.5: Outcome and Covarites |
|  | **Assignment procedures** | | | | |  | **Assignment procedures** | |  |  |
|  | c | Report that eligible individuals would be randomly assigned to treatment strategies and may be aware of their treatment allocation. | | | |  | c | Describe how assignment to treatment strategies was operationalized with the data. |  | p.6-8 Statistical Analysis |
|  | **Follow-up** | | | | |  | **Follow-up** | |  |  |
|  | d | Clarify that follow-up would start at time of assignment to the treatment strategies. Specify when follow-up would end. | | | |  | d | Clarify that follow-up starts at the time individuals were assigned to the treatment strategies. Describe how the end of follow-up was operationalized with the data. |  | p.6-8 Statistical Analysis |
|  | **Outcomes** | | | | |  | **Outcomes** | |  |  |
|  | e | Describe the outcomes. | | | |  | e | Describe how the outcomes were operationalized with the data. |  | p. 5-6 Outcomes and Covariates |
|  | **Causal contrasts** | | | | |  | **Causal contrasts** | |  |  |
|  | f | Describe the causal contrasts of interest, including effect measures. | | | |  | f | Describe how the causal contrasts were operationalized with the data, including effect measures. |  | p.6-8 Statistical Analysis |
|  | **Identifying assumptions** | | | | |  | **Identifying assumptions** | |  |  |
|  | g | Describe assumptions that would be made to identify each causal estimand. Describe the variables, if any, related to these assumptions. | | | |  | g.i | For each causal estimand, describe assumptions made to identify it, including assumptions regarding baseline confounding due to lack of randomization. |  | p.6 Statistical Analysis |
|  |  |  | | | |  | g.ii | Describe how the variables related to these assumptions were operationalized with the data |  | p. 5-6 Outcomes and Covariates |
|  | **Data analysis plan** | | | | |  | **Data analysis plan** | |  |  |
|  | h | For each causal estimand, describe the data analysis procedures and any associated statistical modelling assumptions, including approaches for handling missing data. | | | |  | h.i | For each causal estimand, describe the data analysis procedures and any associated statistical modelling assumptions, including approaches for handling missing data. |  | p.6-8 Statistical Analysis |
|  |  |  | | | |  | h.ii | For each causal estimand, describe any additional analyses conducted to assess the sensitivity of the results to the choice of operationalizations, assumptions and analysis. |  | Figure S5 & S7 |
| **Results** | | | | | | | | | | |
| **8** | Participant selection | | | | Report numbers of individuals assessed for eligibility, eligible, and assigned to each treatment strategy. A flow diagram is strongly recommended. | | | | p.9 Study population | |
| **9** | Baseline data | | | | Describe the distribution of characteristics of individuals at baseline, by treatment strategy. | | | | Table 1 | |
| **10** | Follow-up | | | | Summarize length of follow-up and describe reasons for end of follow-up for each treatment strategy and causal contrast. | | | | p.10 (Always-survivors selection) | |
| **11** | Missing data | | | | Describe the frequency of missing data in all variables, by treatment strategy when applicable. | | | | Not Applicable | |
| **12** | Outcomes | | | | Describe the frequency or distribution of each outcome, by treatment strategy. | | | | Table 1 | |
| **13** | Effect estimates | | | | Report the effect estimates for each causal contrast with corresponding measures of precision, including both absolute and relative measures of effect, when applicable. | | | | p.9-11, Table 2 | |
| **14** | Additional analyses | | | | Report results of all analyses to assess the sensitivity of the estimates to choices in operationalizations, assumptions and analysis. | | | | Supplementary material | |
| **Discussion** | | | | | | | | | | |
| **15** | Interpretation | | Provide an interpretation of the key findings. | | | | | | p.12-13 | |
| **16** | Limitations | | Discuss the limitations of the study considering differences between the target trial and its emulation and the plausibility of assumptions, including assumptions regarding baseline confounding due to lack of randomization. | | | | | | p.14 | |
| **Other information** | | | | | | | | | | |
| **17** | Ethics | | | | Provide the institutional research board or ethics committee that approved the study and approval numbers, if relevant. | | | | Not Applicable | |
| **18** | Registration | | | | State whether, when and where the study protocol was registered. | | | | Not Applicable | |
| **19** | Sharing of study materials | | | | Provide information on whether data, analytic code and/or other materials are accessible, and where and how they can be accessed. | | | | p.19 | |
| **20** | Funding sources | | | | Provide the sources of funding and detail the role of the funders in the design, conduct and reporting of the study. | | | | p.16 | |
| **21** | Conflicts of interest | | | | State any conflicts of interest and financial disclosures for all authors. | | | | p.18 | |

© 2025 Cashin et al. This is an Open Access article distributed under the terms of the Creative Commons Attribution-NoDerivatives License (CC BY-ND 4.0), which permits redistribution, commercial and non-commercial, provided the work is passed along unchanged and in whole, with credit to the original author(s

**Table S2: Patients number by EGFr domain before and after matching**

| *EGFr*  *Domain* | *1* | *2* | *3* | *4* | *5* | *6* | *7* | *8* | *9* | *10* | *11* | *12* | *13* | *14* | *15* | *16* | *17* | *18* | *19* | *20* | *21* | *22* | *23* | *24* | *25* | *26* | *27* | *28* | *29* | *30* | *31* | *32* | *33* | *34* |
| --- | --- | --- | --- | --- | --- | --- | --- | --- | --- | --- | --- | --- | --- | --- | --- | --- | --- | --- | --- | --- | --- | --- | --- | --- | --- | --- | --- | --- | --- | --- | --- | --- | --- | --- |
| *Patients number before matching* | *11* | *36* | *63* | *72* | *21* | *7* | *-* | *1* | *3* | *13* | *1* | *-* | *5* | *14* | *16* | *1* | *-* | *2* | *-* | *-* | *-* | *-* | *-* | *-* | *25* | *7* | *3* | *2* | *-* | *2* | *14* | *4* | *2* | *1* |
| *Patients number after matching* | *6* | *20* | *26* | *44* | *16* | *4* | *-* | *1* | *3* | *13* | *1* | *-* | *5* | *14* | *16* | *1* | *-* | *2* | *-* | *-* | *-* | *-* | *-* | *-* | *25* | *7* | *3* | *2* | *-* | *2* | *14* | *4* | *2* | *1* |

|  |
| --- |

*High-Risk*

|  |
| --- |

*Mid-Risk*

|  |
| --- |

*Low-Risk*

Table S3: Population description of the “always survivors” group at 2 years after first stroke

| **Variables** | **1-6 (n= 116)** | **7-37 (n=116)** | **P-value** |
| --- | --- | --- | --- |
| Male gender (n (%)) | 57 (49%) | 55 (47%) | 0.896 |
| Age at first stroke *(Median (Q1-Q3))* | 54.83 (47.77 – 61.89) | 55.07 (47.69 - 62.45) | 0.895 |
| Nb years of education *(Median (Q1-Q3))* | 10.0 (9.0 - 14.0) | 10.0 (9.0 - 14.0) | 0.352 |
| Hypertension (n (%)) | 62 (53%) | 63 (54%) | 0.896 |
| Hypercholesterolemia (n (%)) | 57 (49%) | 55 (47%) | 0.866 |
| Smoking (n (%)) | 53 (46%) | 65 (56%) | > 0.999 |
| Alcohol (n (%)) | 67 (58%) | 65 (56%) | 0.895 |

**Table S4: Population description of the “always survivors” group after 5 years after the first stroke**

| **Variables** | **1-6 (n= 105)** | **7-37 (n=111)** | **P-value** |
| --- | --- | --- | --- |
| Male gender (n (%)) | 51 (49%) | 50 (45%) | 0.683 |
| Age at first stroke *(Median (Q1-Q3))* | 53.74 (46.96 - 60.52) | 54.47 (47.43 – 60.52) | 0.605 |
| Nb years of education *(Median (Q1-Q3))* | 10.0 (9.0 - 14.0) | 10.0 (9.0 - 14.0) | 0.435 |
| Hypertension (n (%)) | 57 (54%) | 60 (54%) | 0.582 |
| Hypercholesterolemia (n (%)) | 53.74 (46.96-60.52) | 54.47 (47.43– 60.52) | >0.999 |
| Smoking (n (%)) | 60 (57%) | 61 (55%) | > 0.999 |
| Alcohol (n (%)) | 60 (57%) | 50 (45%) | 0.785 |

Table S5: Population description of always survivors at 10 years after first stroke

| **Variables** | **1-6 (n= 88)** | **7-37 (n=108)** | **P-value** |
| --- | --- | --- | --- |
| Male gender (n (%)) | 51 (49%) | 49 (45%) | > 0.999 |
| Age at first stroke *(Median (Q1-Q3))* | 51.99 (45.55 - 58.43) | 53.88 (47.20 – 60.56) | 0.178 |
| Nb years of education *(Median (Q1-Q3))* | 10.0 (9.0 - 15.0) | 10.5 (9.0 - 14.0) | 0.646 |
| Hypertension (n (%)) | 46 (52%) | 59 (55%) | > 0.999 |
| Hypercholesterolemia (n (%)) | 51.99 (45.55-58.43) | 53.88 (47.20– 60.56) | 0.178 |
| Smoking (n (%)) | 37 (42%) | 49 (45%) | > 0.999 |
| Alcohol (n (%)) | 47 (53%) | 59 (55%) | 0.785 |

Table S6: Population description of always survivors at 15 years after first stroke

| **Variables** | **1-6 (n= 63)** | **7-37 (n=91)** | **P-value** |
| --- | --- | --- | --- |
| Male gender (n (%)) | 26 (41%) | 41 (45%) | 0.741 |
| Age at first stroke *(Median (Q1-Q3))* | 49.06 (43.25 - 54.86) | 51.69 (45.90 – 57.49) | 0.178 |
| Nb years of education *(Median (Q1-Q3))* | 11.5 (9.0 - 15.0) | 10.2 (9.0 - 14.0) | 0.186 |
| Hypertension (n (%)) | 23 (37%) | 44 (48%) | 0.745 |
| Hypercholesterolemia (n (%)) | 32 (51%) | 49 (54%) | 0.178 |
| Smoking (n (%)) | 42 (46%) | 25 (40%) | 0.745 |
| Alcohol (n (%)) | 42 (46%) | 25 (40%) | 0.506 |

**Table S7: Comparison of baseline characteristics between patients left censored or not for mRS>3**

| **Variables** | **Censored**  **(n= 32)** | **Non-censored**  **(n=293)** | **P-value** |
| --- | --- | --- | --- |
| Male gender (n (%)) | 13 (41%) | 135 (41%) | 0.581 |
| Age at first stroke *(Median (Q1-Q3))* | 49.06 (43.25 - 54.86) | 51.69 (45.90 – 57.49) | 0.178 |
| Nb years of education *(Median (Q1-Q3))* | 9.0 (6.0 – 12.5) | 11.3 (9.0 - 14.0) | 0.177 |
| Hypertension (n (%)) | 9 (28%) | 91 (31%) | 0.842 |
| Hypercholesterolemia (n (%)) | 20 (63%) | 129 (44%) | 0.061 |
| Smoking (n (%)) | 12 (38%) | 151 (52%) | 0.141 |
| Alcohol (n (%)) | 15 (47%) | 166 (57%) | 0.350 |

**Table S8: Sensitivity analysis for censoring by interval for mRS analysis: RMST analysis using the start and the middle of the interval as event date**

| **Years to predict** | **RMST 1-6 mutation** | **RMST 7-34 mutation** | **Difference** | **P.value** |
| --- | --- | --- | --- | --- |
| **Start of the censored interval** |  |  |  |  |
| 2 years | 1.91 [1.84-1.97] | 1..98 [1.95-2.01] | 0.07 | 0.052 |
| **5 years** | **4.66 [4.45-4.86]** | **4.94 [4.85-5.03]** | **0.28** | **0.014** |
| **10 years** | **8.78 [8.21-9.86]** | **9.71 [9.43-9.98]** | **0.93** | **0.004** |
| 15 years | 12.96 [11.83-14.09] | 13.96 [13.27-14.65] | 1.00 | 0.138 |
| **Middle of the censored interval** |  |  |  |  |
| 2 years | 1.94 [1.89-1.99] | 1.97 [1.94-2.01] | 0.03 | 0.25 |
| **5 years** | **4.73 [4.55-4.95]** | **4.94 [4.86 – 5.02** | **0.21** | **0.05** |
| **10 years** | **8.92 [8.40-9.46]** | **9.73 [9.47-10.00]** | **0.81** | **0.01** |
| 15 years | 13.10 [12.05-14.16] | 14.00 [13.32-14.68] | 0.89 | 0.16 |

*Bold values indicate statistically significant results (p < 0.05)*

Table S9 : RMST Comparison of Death Between EGFR Mutation (High-Risk vs Mid-Risk) at 5, 10, and 15 Years Post-First Stroke; and of Second Stroke and mRS >3, between EGFR Mutation (High-Risk vs Mid-Risk) at 2, 5, 10, and 15 Years Post-First Stroke in Distinct “always-survivor” Subgroups (i.e., patients surviving at least to each timepoint)

| **Years to predict** | **Nb « always survivors »** | **RMST High-Risk mutation** | **RMST Mid-Risk mutation** | **Difference** | **P.value** |
| --- | --- | --- | --- | --- | --- |
| **Death** |  |  |  |  |  |
| 5 years | - | 4.88 [4.84-5.00] | 4.95 [4.91 – 5.01] | -0.08 | 0.397 |
| **10 years** | **-** | **9.16 [8.72-9.69]** | **9.72 [9.40-10.03]** | **-0.56** | **0.042** |
| **15 years** | **-** | **12.26 [11.34-13.17]** | **14.17 [13.53-14.81]** | **-1.90** | **0.001** |
| **Second stroke** |  |  |  |  |  |
| 2 years | 166 | 1.84 [1.76-1.93] | 1.70 [1.56 - 1.84] | 0.14 | 0.090 |
| 5 years | 160 | 3.98 [3.621-4.347] | 3.83 [3.426;4.242] | 0.15 | 0.590 |
| 10 years | 149 | 6.67 [5.721-7.623] | 6.50 [5.581-7.415] | 0.17 | 0.796 |
| 15 years | 112 | 8.98 [7.140-10.820] | 8.09 [7.39-10.08] | 0.90 | 0.467 |
| **Rankin > 3** |  |  |  |  |  |
| 2 years | 166 | 0.049 [-0.01-0.11] | 0 [0.00-0.00] | 0.05 | 0.094 |
| 5 years | 160 | 4.76 [4.57-4.95] | 4.94 [4.86-5.03] | 0.18 | 0.087 |
| **10 years** | **149** | **8.67 [8.03-9.30]** | **9.79 [9.50-10.08]** | **1.12** | **0.002** |
| 15 years | 112 | 13.36 [12.02-14.70] | 14.07 [13.35-14.79] | 0.71 | 0.360 |

*Bold values indicate statistically significant results (p < 0.05)*

Figure S1: Principal stratification classification at 2 years after first stroke


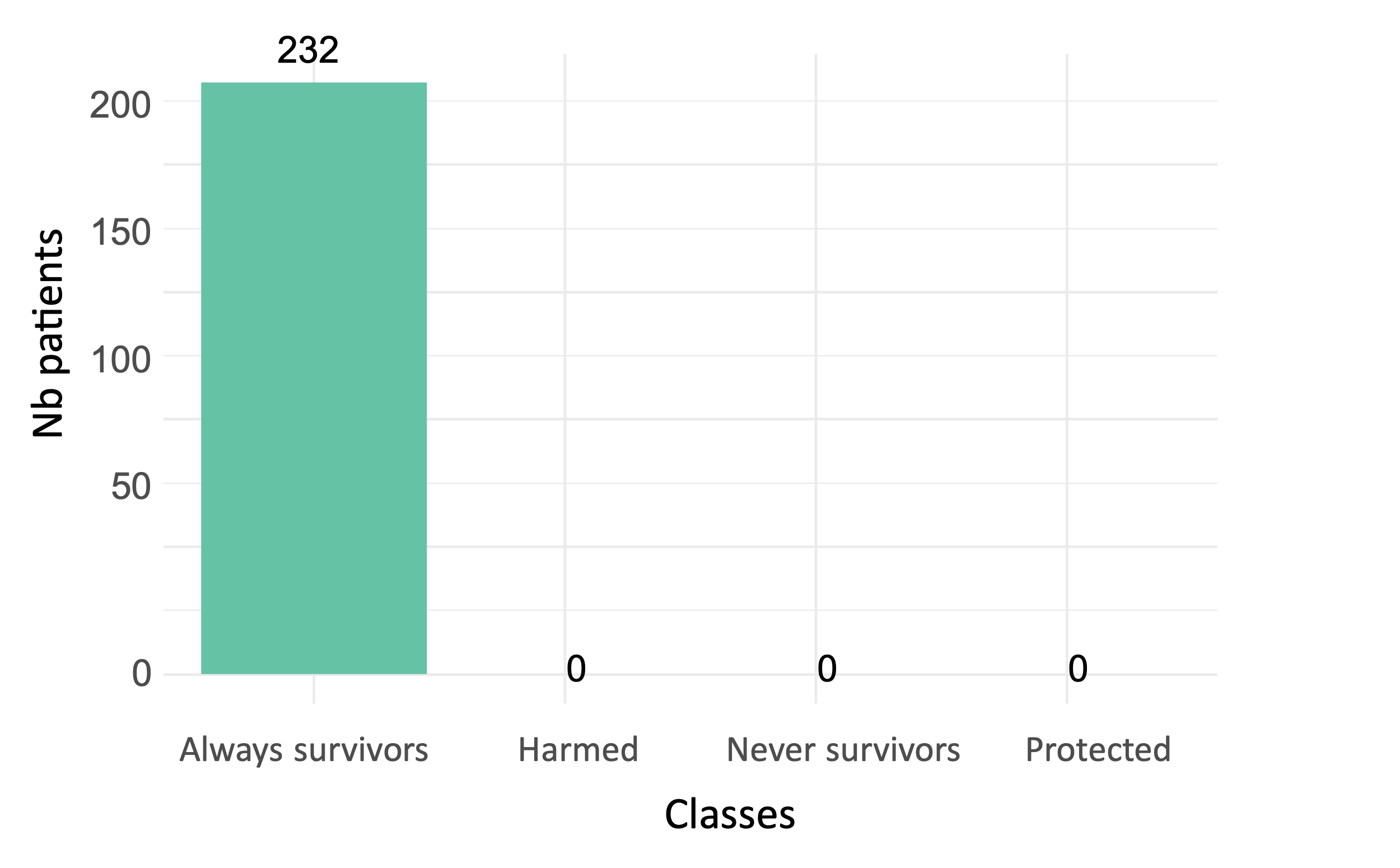


Figure S2: Principal stratification classification at 5 years after first stroke


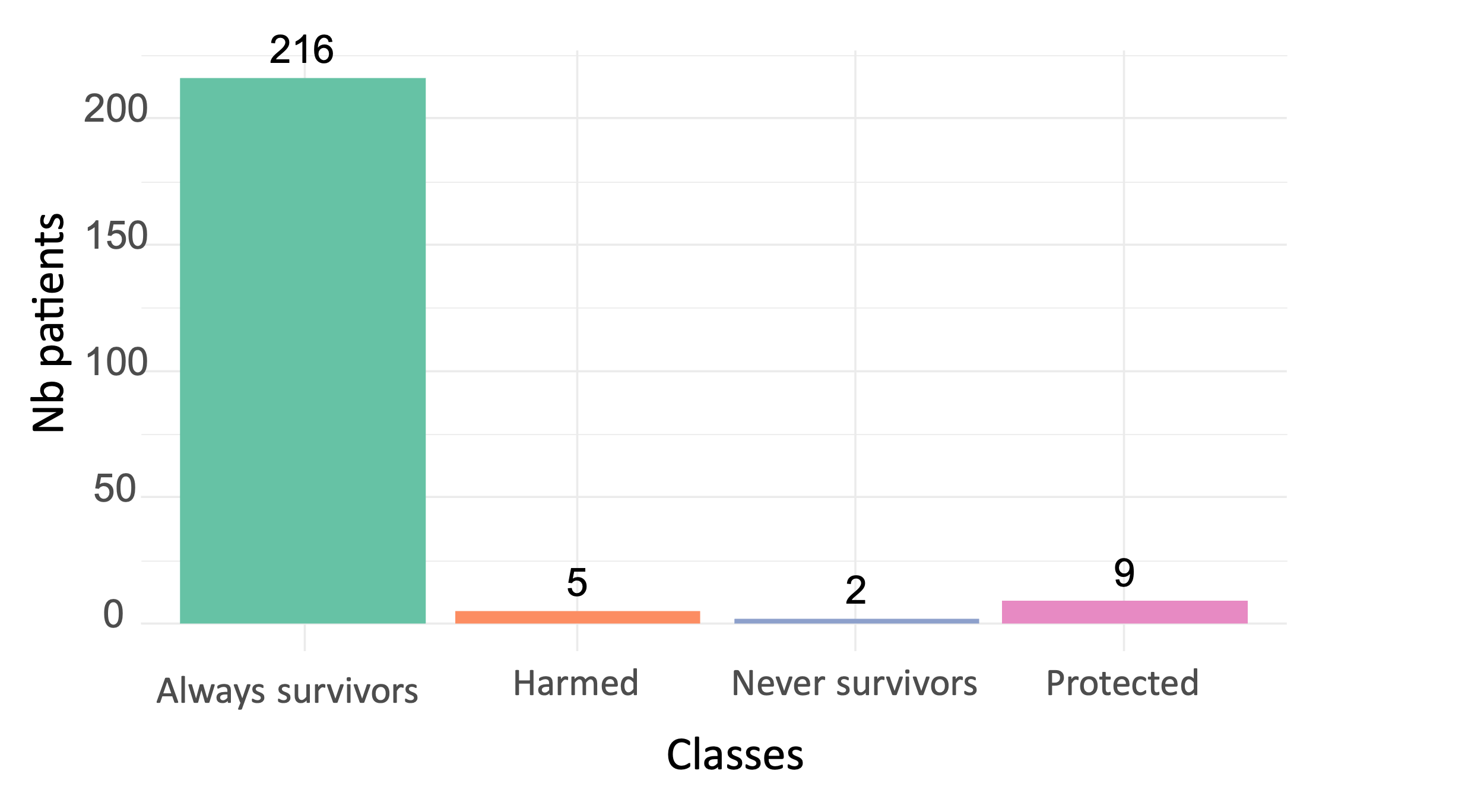


Figure S3: Principal stratification classification at 10 years after the first stroke


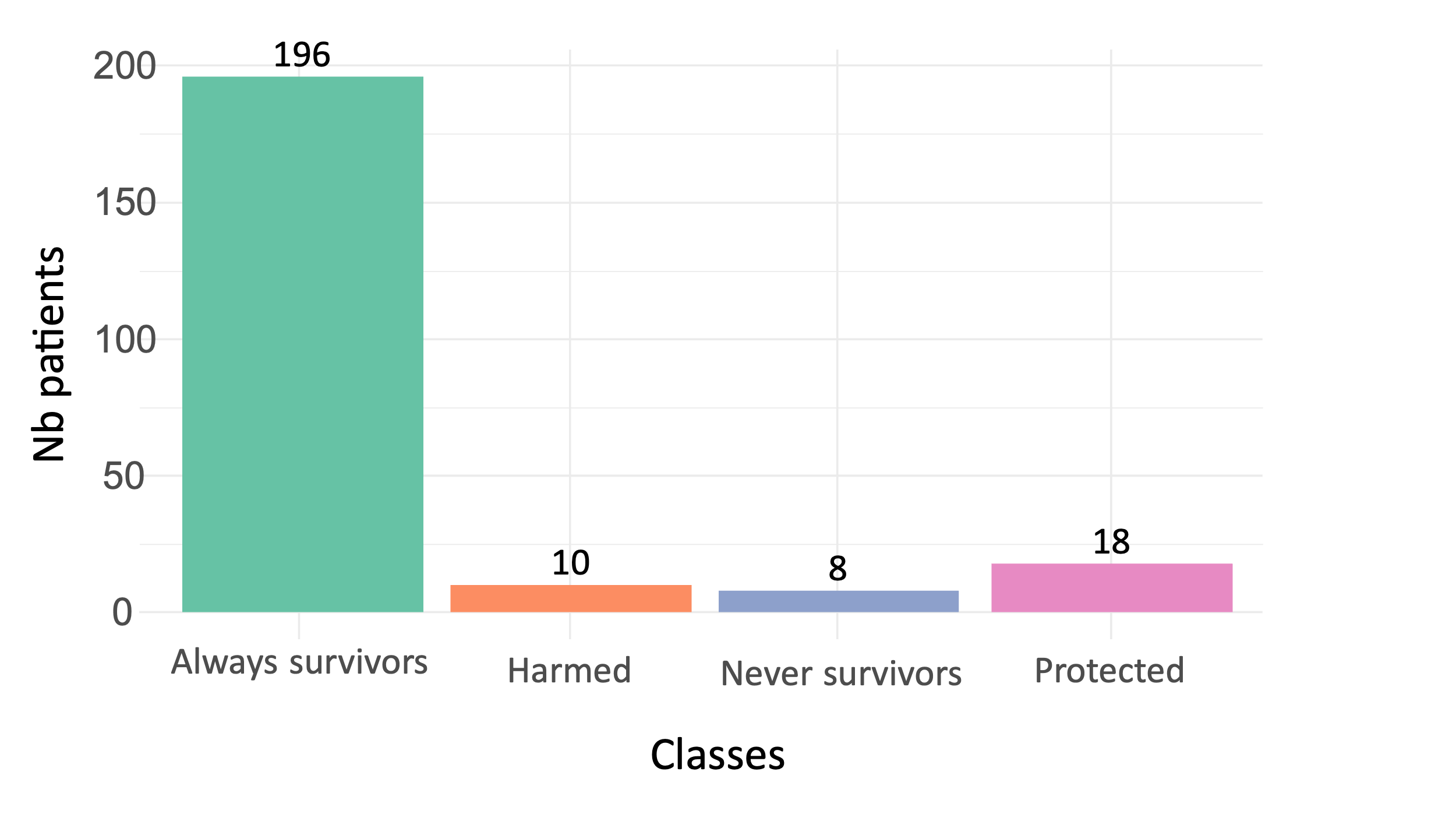


Figure S4: Principal stratification classification at 15 years after first stroke


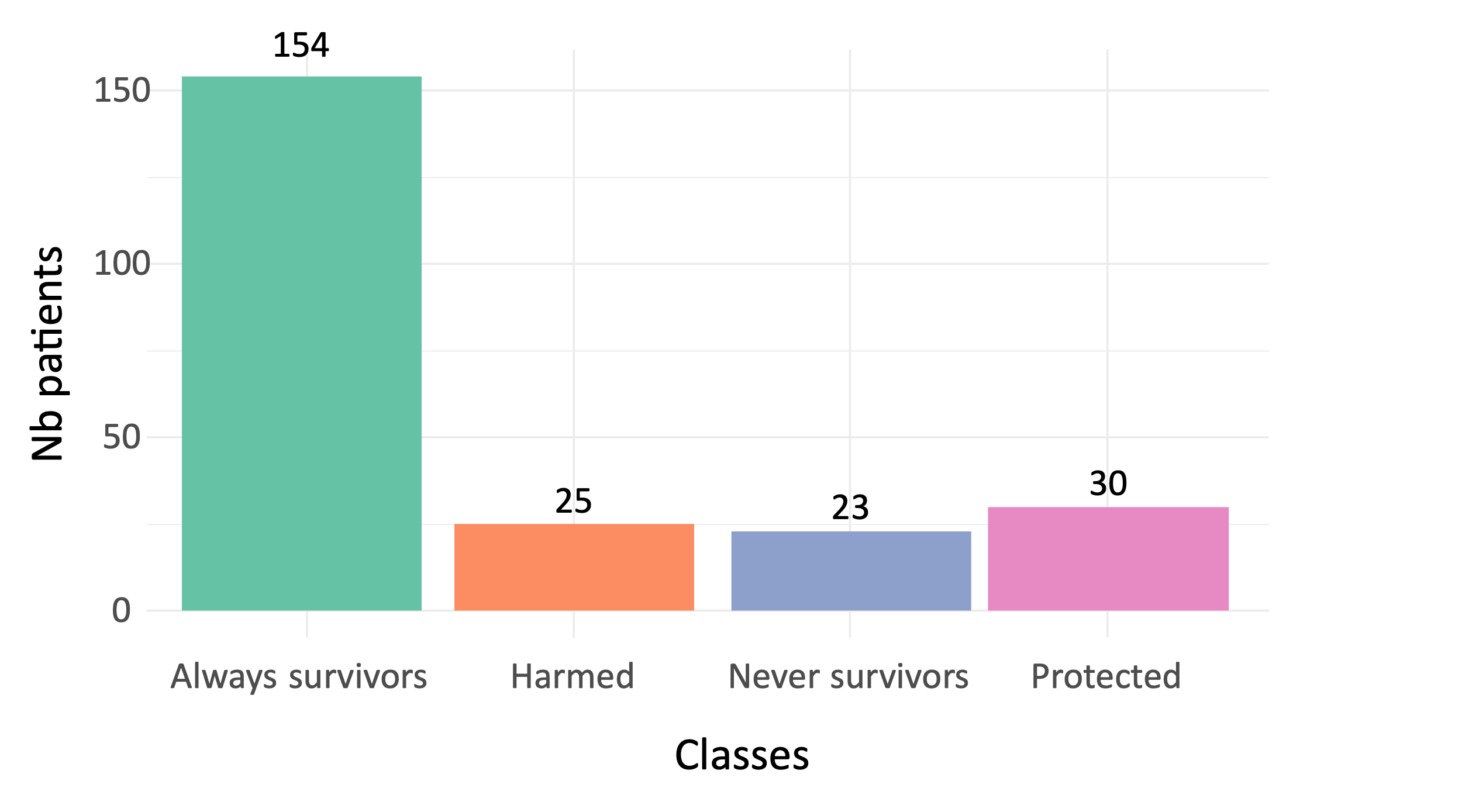


Figure S5: Sensitivity analysis: Adjusted Kaplan-Meier estimates of stroke-free survival from 2 (A), 5 (B), 10 (C), and 15 (D) years after first stroke, in “always-survivor” subgroups, classifying as deaths (death prioritized over stroke), stratified by EGFR mutation domain (1–6 vs. 7–34)


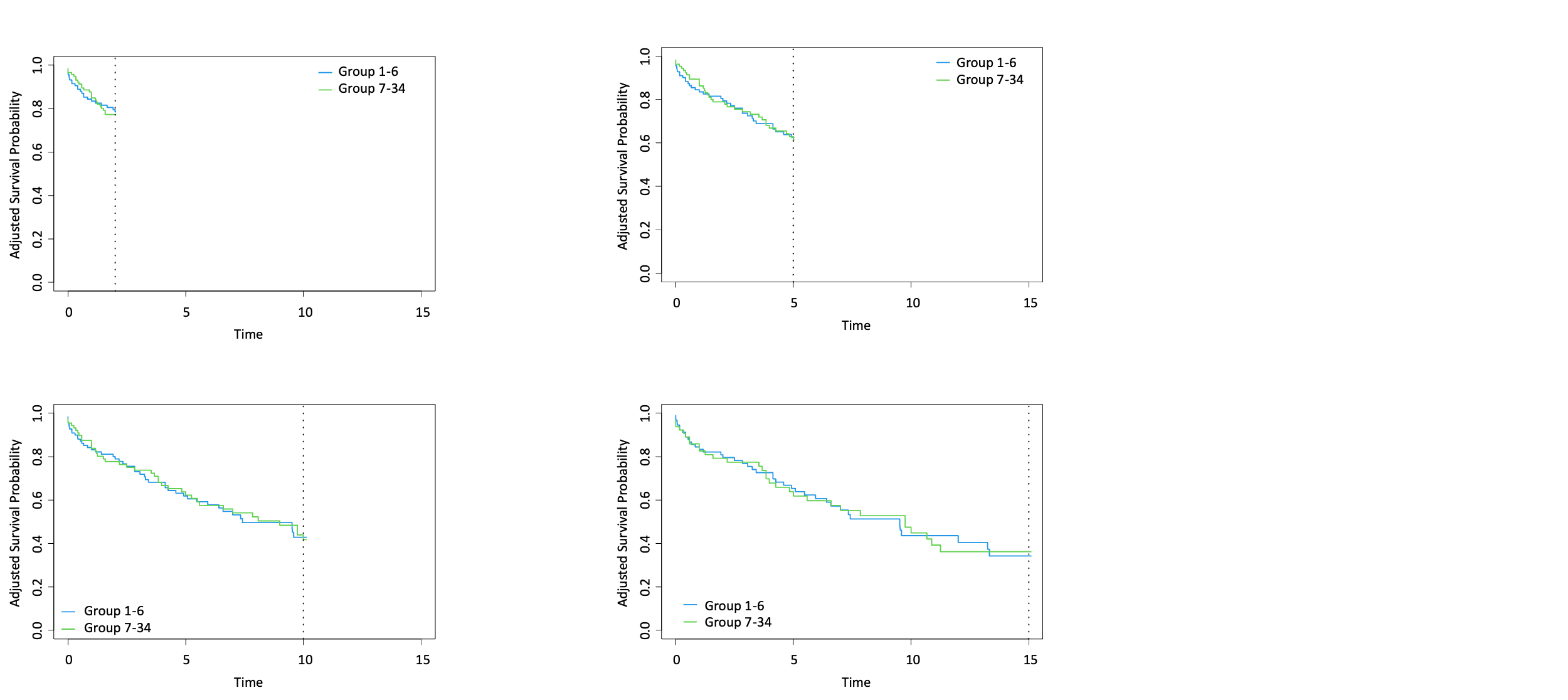


**A**

**C**

**B**

**D**

**Figure S6: Length of the interval censoring for Rankin analysis, depending on baseline covariates**


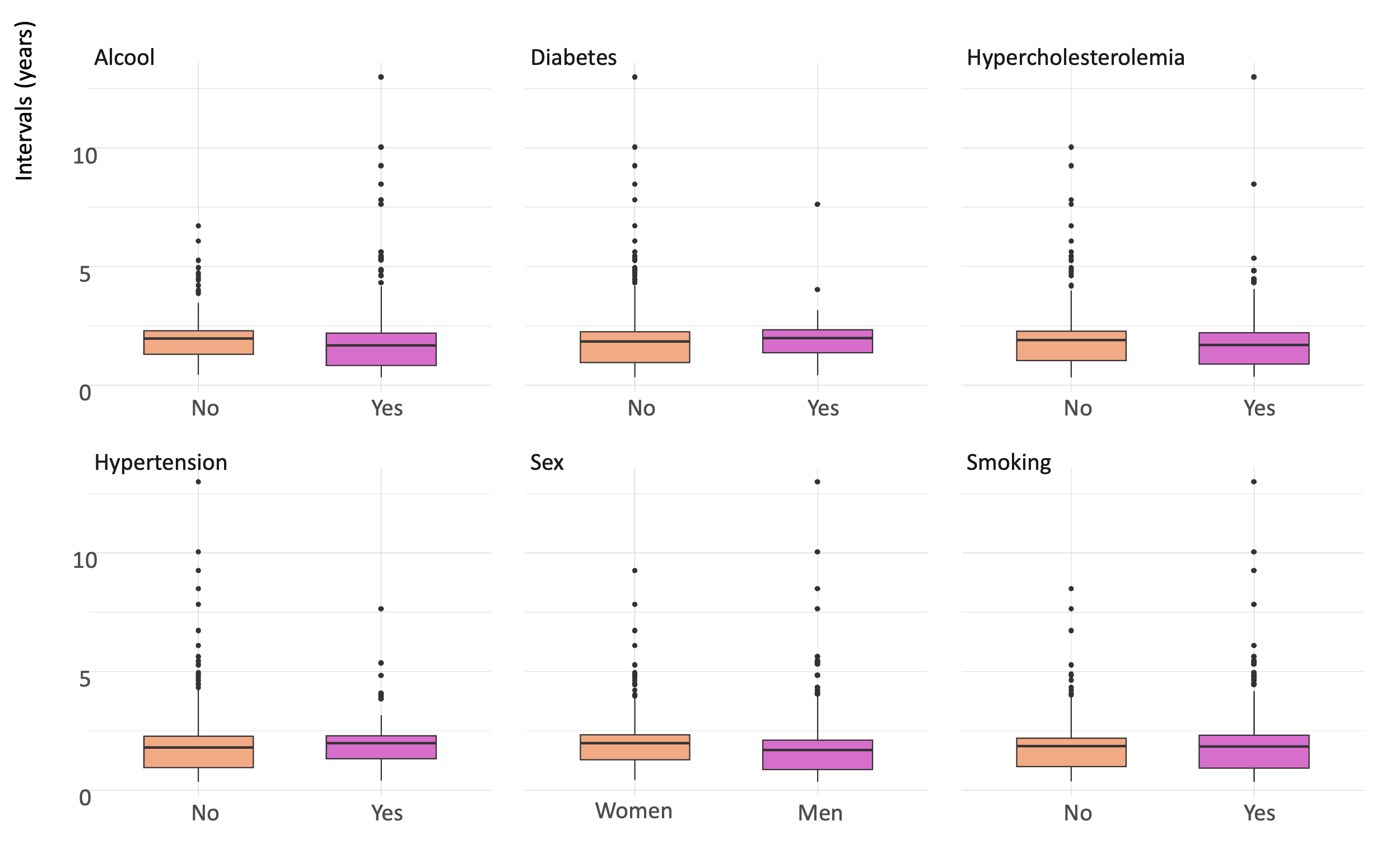

Supplement: Supplementary file 1 — Figure S1: Principal stratification classification at 2 years after first stroke. Figure S2: Principal stratification classification at 5 years after first stroke. Figure S3: Principal stratification classification at 10 years after the first stroke. Figure S4: Principal stratification classification at 15 years after first stroke. Figure S5: Sensitivity analysis: Adjusted Kaplan–Meier estimates of stroke‐free survival from 2 (A), 5 (B), 10 (C), and 15 (D) years after first stroke, in “always‐survivor” subgroups, classifying as deaths (death prioritized over stroke), stratified by EGFR mutation domain (1–6 vs. 7–34). Figure S6: Length of the interval censoring for Rankin analysis, depending on baseline covariates. Table S1: TARGET Guideline checklist. Table S2: Patients number by EGFr domain before and after matching. Table S3: Population description of the “always survivors” group at 2 years after first stroke. Table S4: Population description of the “always survivors” group after 5 years after the first stroke. Table S5: Population description of always survivors at 10 years after first stroke. Table S6: Population description of always survivors at 15 years after first stroke. Table S7: Comparison of baseline characteristics between patients left censored or not for mRS > 3. Table S8: Sensitivity analysis for censoring by interval for mRS analysis: RMST analysis using the start and the middle of the interval as event date. Table S9: RMST Comparison of Death Between EGFR Mutation (High‐Risk vs. Mid‐Risk) at 5, 10, and 15 Years Post‐First Stroke; and of Second Stroke and mRS > 3, between EGFR Mutation (High‐Risk vs. Mid‐Risk) at 2, 5, 10, and 15 Years Post‐First Stroke in Distinct “always‐survivor” Subgroups (i.e., patients surviving at least to each timepoint). [file ACN3-9999-0-s001.docx]
